# Thermodynamic feasibility of shipboard conversion of marine plastics to blue diesel for self-powered ocean cleanup

**DOI:** 10.1073/pnas.2107250118

**Published:** 2021-11-01

**Authors:** Elizabeth R. Belden, Nikolaos K. Kazantzis, Christopher M. Reddy, Hauke Kite-Powell, Michael T. Timko, Eduardo Italiani, Dudley R. Herschbach

**Affiliations:** ^a^Department of Chemical Engineering, Worcester Polytechnic Institute, Worcester, MA 01069;; ^b^Department of Marine Chemistry and Geochemistry, Woods Hole Oceanographic Institution, Woods Hole, MA, 02543;; ^c^Marine Policy Center, Woods Hole Oceanographic Institution, Woods Hole, MA 02543;; ^d^Department of Chemistry and Chemical Biology, Harvard University, Cambridge, MA 02138

**Keywords:** ocean plastic, hydrothermal liquefaction, exergy analysis, Monte Carlo simulation

## Abstract

Collecting and removing ocean plastics can mitigate their environmental impacts; however, ocean cleanup will be a complex and energy-intensive operation that has not been fully evaluated. This work examines the thermodynamic feasibility and subsequent implications of hydrothermally converting this waste into a fuel to enable self-powered cleanup. A comprehensive probabilistic exergy analysis demonstrates that hydrothermal liquefaction has potential to generate sufficient energy to power both the process and the ship performing the cleanup. Self-powered cleanup reduces the number of roundtrips to port of a waste-laden ship, eliminating the need for fossil fuel use for most plastic concentrations. Several cleanup scenarios are modeled for the Great Pacific Garbage Patch (GPGP), corresponding to 230 t to 11,500 t of plastic removed yearly; the range corresponds to uncertainty in the surface concentration of plastics in the GPGP. Estimated cleanup times depends mainly on the number of booms that can be deployed in the GPGP without sacrificing collection efficiency. Self-powered cleanup may be a viable approach for removal of plastics from the ocean, and gaps in our understanding of GPGP characteristics should be addressed to reduce uncertainty.

## Significance

Plastic waste accumulating in the world's oceans forms massive “plastic islands” in the oceanic gyres. Removing the plastic offers an opportunity to restore our oceans to a more pristine state. To clean the gyres, ships must collect and store the plastic before transporting it to port, often thousands of kilometers away. Instead, ocean plastic waste can be converted into fuel shipboard, for example, using hydrothermal liquefaction (HTL), which depolymerizes plastics at high temperature (300 °C to 550 °C) and high pressure (250 bar to 300 bar). The resulting depolymerization products, termed “blue diesel,” have the potential for self-powered cleanup. The objective of this work is evaluating the thermodynamic feasibility of this scheme and its implications on cleanup.

An estimated 4.8 million to 12.7 million tons of plastic enter the ocean each year, distributing widely across the ocean’s surface and water column, settling into sediments, and accumulating in marine life (1–3). Numerous studies have shown that plastics contribute to significant damages to marine life and birds, therefore motivating introduction of effective mitigation and removal measures (4). Reducing or eliminating the amount of plastic waste generated is critically important, especially when the current loading may persist for years to even decades (1, 5, 6).

As a highly visible part of an integrated approach for removing plastics from the environment (1, 5, 6), efforts are underway to collect oceanic plastic from accumulation zones in gyres formed by ocean currents (3, 7). Present approaches to remove plastic from the open ocean utilize a ship that must store plastic on board until it returns to port, often thousands of kilometers away, to unload the plastic, refuel, and resupply.

Optimistic evaluation of cleanup time using the harvest–return approach indicates that at least 50 y will be required for full plastic removal (7), with an annual cost of $36.2 million (8); more conservative estimates suggest that partial removal will require more than 130 y (7, 9). Cleanup times of decades mean that environmental degradation may have already reduced the existing plastics to microscopic and smaller forms that can no longer be harvested before cleanup is completed (1, 4, 9). These considerations underscore the massive challenge of removing plastics from the ocean and naturally raise the following question: Can any approach remove plastics from the ocean faster than they degrade?

Some current plastic removal strategies involve accumulation via a system of booms, consisting of semicircular buoys fit with a fine mesh extending below the ocean surface (7, 10). These booms are positioned so that prevailing currents bring plastic to the boom, where it then accumulates. The currently envisioned approach is for a ship to steam to the boom system, collect plastic, and then return to port to offload and refuel before resuming collection activities.

The time required for recovering plastics could be reduced if return trips to refuel and unload plastic were eliminated. Indeed, the harvested plastic has an energy density similar to hydrocarbon fuels; harnessing this energy to power the ship could thereby eliminate the need to refuel or unload plastic from the ship, reducing fossil fuel usage and potentially cleanup times.

Self-powered harvesting may provide a way to accomplish cleanup using the passive boom collection approach at timescales less than environmental degradation. Unfortunately, cleanup itself is a moving target, as technology improves (7) and especially as plastic continues to accumulate. What is required, therefore, is a framework to evaluate the impact of self-powered harvesting on cleanup time and fuel usage. The framework can then be updated as more data becomes available.

To be valuable, the cleanup framework must be reducible to practice using actual technology. A viable technology for converting plastics into a usable fuel is hydrothermal liquefaction (HTL), which utilizes high temperature (300 °C to 550 °C) and high pressure (250 bar to 300 bar) to transform plastics into monomers and other small molecules suitable as fuels (11–13). Oil yields from HTL are typically >90% even in the absence of catalysts and, unlike pyrolysis, yields of solid byproducts—which would need to be stored or burned in a special combustor—are less than 5% (11–13), thus conferring certain comparative advantages to HTL. Ideally, a vessel equipped with an HTL-based plastic conversion system could fuel itself, creating its fuel from recovered materials. The result could be termed “blue diesel,” to reference its marine origin and in contrast with both traditional marine diesel and “green diesel,” derived from land-based renewable resources (14).

To make the HTL approach feasible, the work produced from the plastic must exceed that required by the process and, ideally, the ship’s engines so that fuel can be stockpiled during collection for later use. Exergy analysis provides a framework to determine the maximum amount of work that a complex process is capable of producing without violating the fundamental laws of thermodynamics (15). The reliability of an exergy analysis depends on the reliability of the data it uses as inputs, and key parameters describing HTL performance and ocean surface plastic concentration are currently not known with certainty. A rigorous and statistically meaningful analysis of shipboard plastic processing must therefore integrate uncertainty (16). Here, the Monte Carlo (MC) simulation method, which has proven its usefulness for similar types of analyses, is an appropriate tool for handling the uncertainties inherent in the current application (17) and allows for the integration of new information and data as further study of oceanic surface plastic is completed.

Accordingly, the thermodynamic performance of a shipboard HTL process was evaluated to determine whether (and when) the process could provide sufficient energy to power itself plus the ship. A framework was then developed to evaluate the implications of shipboard plastic conversion on fuel use and cleanup times. The results provide valuable insight into the potential use of shipboard conversion technologies for accelerating removal of plastics from the ocean, and the framework should prove useful for guiding future work in this area.

## Results

### Thermodynamic Analysis of Shipboard HTL Plastic Conversion.

**Fig. 1.**
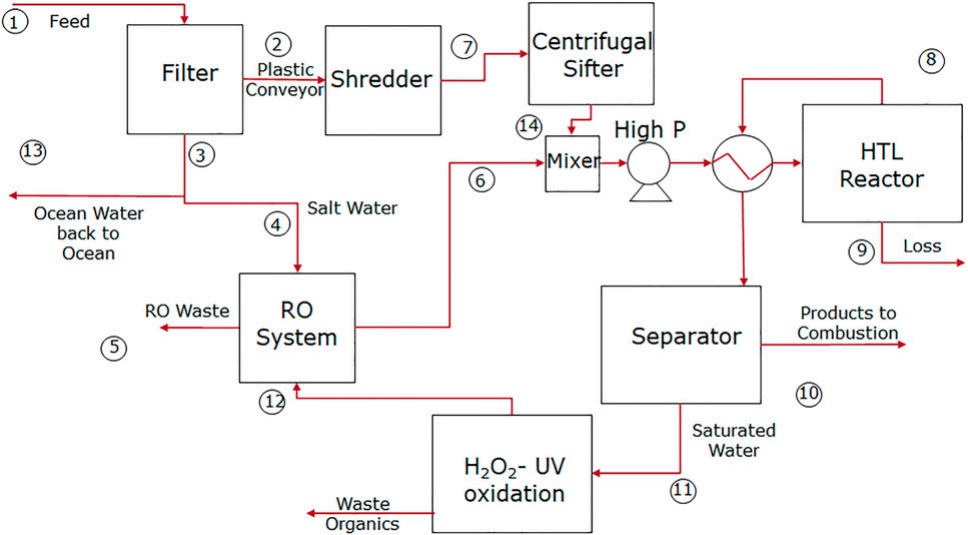
Conceptual design of a shipboard HTL-based process for converting ocean-borne plastics into usable fuel: process flow diagram. The entire process is designed to fit within a standard 20-ft shipping container.

The first step was a thermodynamic analysis of a realistically configured plastic conversion process. Fig. 1 provides a schematic of the proposed plastic conversion system that includes components to collect and shred the plastic, remove salts and other impurities, and convert the plastic feed into blue diesel, a marine plastic–derived fuel with energy density and volatility similar to marine diesel (18). Details can be found in *SI Appendix*, section SI.1.1 and Table SI.1. The entire process is self-contained and can easily fit on a ship, selected here as 40 m—smaller than current vessels in use for the removal of ocean plastics (7), since the ship with on-board conversion does not need to store plastic that it collects.

Thermodynamic performance of the HTL reactor itself is determined by oil yield and heating value obtained at a given reaction temperature. The relationship between oil yield and temperature depends on the composition of the plastic feed and is measured experimentally. Accordingly, simulations were performed for two individual pure plastics commonly found in the ocean (2), polypropylene (PP) and polyethylene (PE), as well as a “mixed feed” based on the typical composition of marine plastics (2:1 PE:PP) (2). The operating temperature, overall conversion, and product yield for each plastic feed were taken from previous work (11–13) and are listed in *SI Appendix*, Tables SI.2 and SI.3 (*SI Appendix*, section SI.1.2). Notably, performance for the PP/PE mixed feed was especially promising, as HTL of this feed resulted in 85% oil yield at a modest temperature (400 °C) and with no solid byproduct.

HTL produces chemical energy in a form usable by the engine. All peripheral equipment detracts from the ideal thermodynamic performance. Parasitic losses for operation of the peripheral equipment required for shredding, heating, pumping, and separations were included in the system-level analysis (see *SI Appendix*, Table SI.1). Performance of the system consisting of both the HTL reactor and the ship itself was evaluated for operation of the ship at either what is termed normal speed or extra slow steaming, a speed that is recommended for minimizing fuel consumption (19).

Published performance, thermodynamic properties, and equipment specifications provide a basis for a comprehensive exergy model of the HTL-based process. However, most of the required parameters are not known with accuracy; projecting them to scale introduces additional uncertainty. Accordingly, the uncertainties of key parameters were included in the exergy model using an MC sampling approach (20).

After testing the effect of several parameters, the six shown in Table 1 were chosen for inclusion in the uncertainty analysis, along with the overall conversions for each plastic (for reaction condition details, see *SI Appendix*, Table SI.2). Table 1 provides justification for the ranges and the associated probability distribution functions selected for the uncertainty quantification of all pertinent model input variables.

**Table 1.** Probability distribution profiles of the uncertain model input variables

Parameter	Uniform probability distribution ranges	Justification
Weight percent plastic in reactor	10 to 30%	Maintain realistic pumping capabilities
Ocean temperature	17 °C to 30 °C	Account for varying ocean temperature based on location and season
Heat exchanger efficiency	50 to 80%	Based on average heat exchanger efficiencies
Engine power	1,800 hp to 2,200 hp	Engine size of an average large fishing trawler with a 10% variance to account for variable weather conditions and engine power draws
Engine efficiency	35 to 40%	Based on current engine technology efficiencies
Heat of combustion variance	0.98 to 1.02	Account for slight variance in selectivity of products and therefore variance in heat of combustion
PP overall conversion to oil	60 to 100%	Optimal literature value with variance (11)
PE overall conversion to oil	60 to 80%	Optimal literature value with variance (12)
PE/PP mixture overall conversion to oil	70 to 90%	Optimal literature value with variance (13)

In most cases, uncertain model input variables are centered on their most probable value following the aforementioned probability distribution profiles. Oil yield is the only exception. For oil yields, the optimal values published in the literature were selected as the maximum point in a uniform MC probability distribution function. This approach is an attempt to account for the effects of additives, aging, and contaminants (21), all of which are expected to decrease oil yield.

Having selected the model input variables and their ranges (Table 1), input uncertainty waspropagated through the aforementioned exergy model to derive a probability distribution profile of exergy outcomes, which can be probabilistically characterized and predict either net exergy consumption or generation zones. Therefore, the derived probability distribution profile and range of possible thermodynamic performance outcomes enables an insightful identification of opportunities and risks in a more robust and nuanced manner than using standard single-point exergy estimates as in conventional approaches.

The MC-based model does not include uncertainty in the loading of plastic on the ocean surface, a key parameter governing performance. Unfortunately, the plastic loading has only been roughly estimated and varies from location to location (2, 5). Accordingly, to account for uncertainty in the plastic loading, we combined the aforementioned MC process simulation with a traditional sensitivity analysis of plastic loading, by simulating the performance over a range of plastic loadings.

**Fig. 2.**
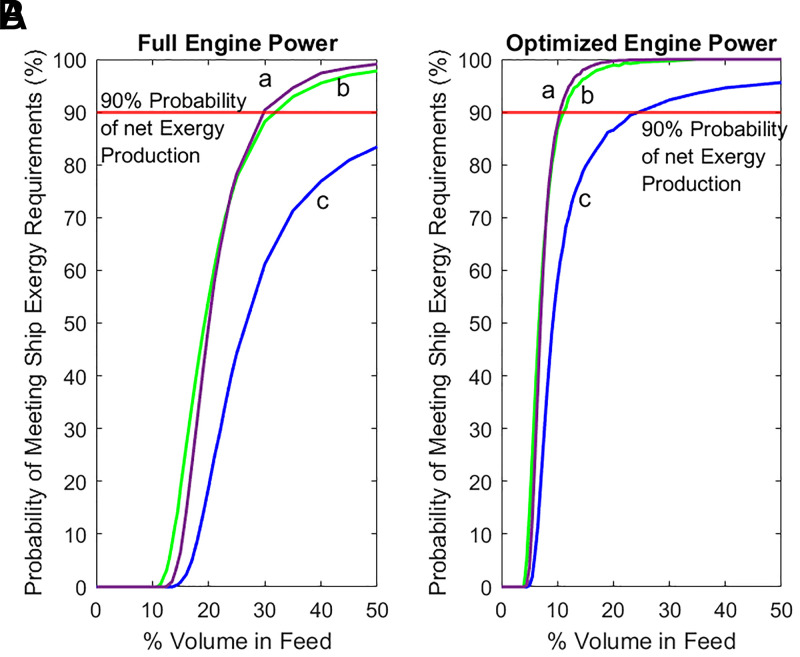
Probability of producing more exergy than is consumed by the combination of the HTL process itself and the ship’s engine for (line a) a 2:1 PE to PP mixture, (line b) PP, and (line c) PE for (*A*) full engine power and (*B*) optimized engine power [1/3 engine power (19)].

Fig. 2 shows the probability of shipboard HTL producing more exergy than the process and the ship itself consume, when the ship travels at full engine power (Fig. 2*A*) and optimized engine power (1/3 engine power) (Fig. 2*B*) to conserve fuel. The temperature required for PE decomposition is greater than for PP even though depolymerization of both feeds results in similar oil yields, thus accounting for the difference in performance predicted for PE compared with PP.

Interestingly, performance profiles for the PP and the PE/PP feeds exhibit similar characteristics to one another, a promising finding given that the PE/PP mixture is most representative of the composition of plastic present in the Great Pacific Garbage Patch (GPGP) (1). Based on previous modeling of the depolymerization of mixtures, the likely explanation for the favorable performance observed for PE/PP is an autocatalytic effect arising from radicals formed by PP pyrolysis (13).

The point in Fig. 2 at which the probability of net exergy production is greater than 90% takes specific importance, as it represents the point where the risk is sufficiently low in the presence of modeled uncertainty (22) (*SI Appendix*, section SI.2 contains further details). Fig. 2*B* shows that the 90% probability point is reached when the ship operates at optimized engine power for all plastic loadings of <25% and <12% for PP and the PE/PP mixture. A plastic loading of 12% is within the range predicted for collection by a boom placed within a gyre (9). However, the break-even point loading is much greater than is expected without a boom, meaning that HTL conversion is not self-powering in the open ocean.

Fig. 2 shows a range in predicted outcomes that is the result of the current level of uncertainties in HTL thermodynamics and conversion rates. Improved data and predictive models will reduce the range of predicted outcomes, thereby derisking investment in the approach. Fig. 2 also suggests that optimizing engine power [1/3 engine power (19)] is necessary for thermodynamically favorable processing of mixed plastic streams.

*SI Appendix*, Fig. SI.1 provides more detail on the exergy consumed by the various subprocesses, taken at the point of >90% probability of producing net exergy. In all cases, the ship itself is the main source of exergy consumption, followed by heating the feed to the HTL reactor.

Pyrolysis was also considered as a possible technology option for self-powered ocean cleanup. Like HTL, pyrolysis is a thermal depolymerization process that yields an oil product that can be used as fuel. Unlike HTL, pyrolysis requires a dry feed, meaning that exergy is required to dry the ocean plastic stream prior to pyrolyzing it. Similarly, pyrolysis produces greater yields of solid byproducts than HTL; these solid byproducts must be stored on board—which reduces one of the main benefits of shipboard conversion—or burned for heat in a dedicated burner. On the other hand, HTL requires preheating of the liquid water feed, only part of which can be recovered from the product stream. Thermodynamic analysis is required to evaluate these trade-offs.

*SI Appendix*, Fig. SI.2 provides a comparison of HTL and pyrolysis for the mixed PE/PP stream. Interestingly, thermodynamic performance profiles of HTL and pyrolysis are very similar to one another, indicating that the differences in drying, heating, and oil yields obtainable by the two processes nearly offset one another (13, 23). Therefore, from a strictly thermodynamic standpoint, either pyrolysis or HTL could be a viable shipboard technology option. That stated, the pyrolysis oil yield is significantly less than the one attained by HTL (65% compared with 85%), meaning that byproduct disposal is much more difficult for pyrolysis than HTL (23). These byproducts consist of gases and chars that will have to be flashed, stored, or burned in solid-compatible combustors. The pyrolysis system footprint will be greater than that required for HTL, to accommodate the dryer required for pyrolysis. These secondary considerations indicate that HTL is the more promising technology option for shipboard conversion; however, pyrolysis remains a viable option should HTL prove difficult to implement.

Scale is an important consideration, and, in addition to the base case, which assumed a flowrate of 3.6 m^3^⋅h^−1^, a second case was also considered for the mixed plastic stream with a flow rate of 36 m^3^⋅h^−1^. The benefit of increasing the exergy output for a ship of a fixed size more than counterbalanced the increased energy requirements of the peripheral equipment, shifting the 90% probability point from 30 vol% to 3.3 vol% (*SI Appendix*, Fig. SI.3). Accordingly, thermodynamic considerations encourage scaling the process as large as practically possible within size, weight, and economic constraints of a given vessel. This suggests a logical extension of the present work: the joint optimization of vessel and process size, and vessel endurance, with detailed considerations of space and energy needs of the crew and all onboard systems.

### A Framework to Evaluate the Implications of Shipboard HTL Conversion on Ocean Cleanup.

The previous section (*Thermodynamic Analysis of Shipboard HTL Plastic Conversion*) predicts that shipboard HTL enables self-powered harvesting of plastics that have first been collected by a boom placed within a natural oceanic gyre. Accordingly, the impact of shipboard HTL conversion was projected within a cleanup framework for a concrete application: estimating the yearly removal of plastics from the GPGP, a gyre located in the central portion of the Pacific Ocean (2).

**Fig. 3.**
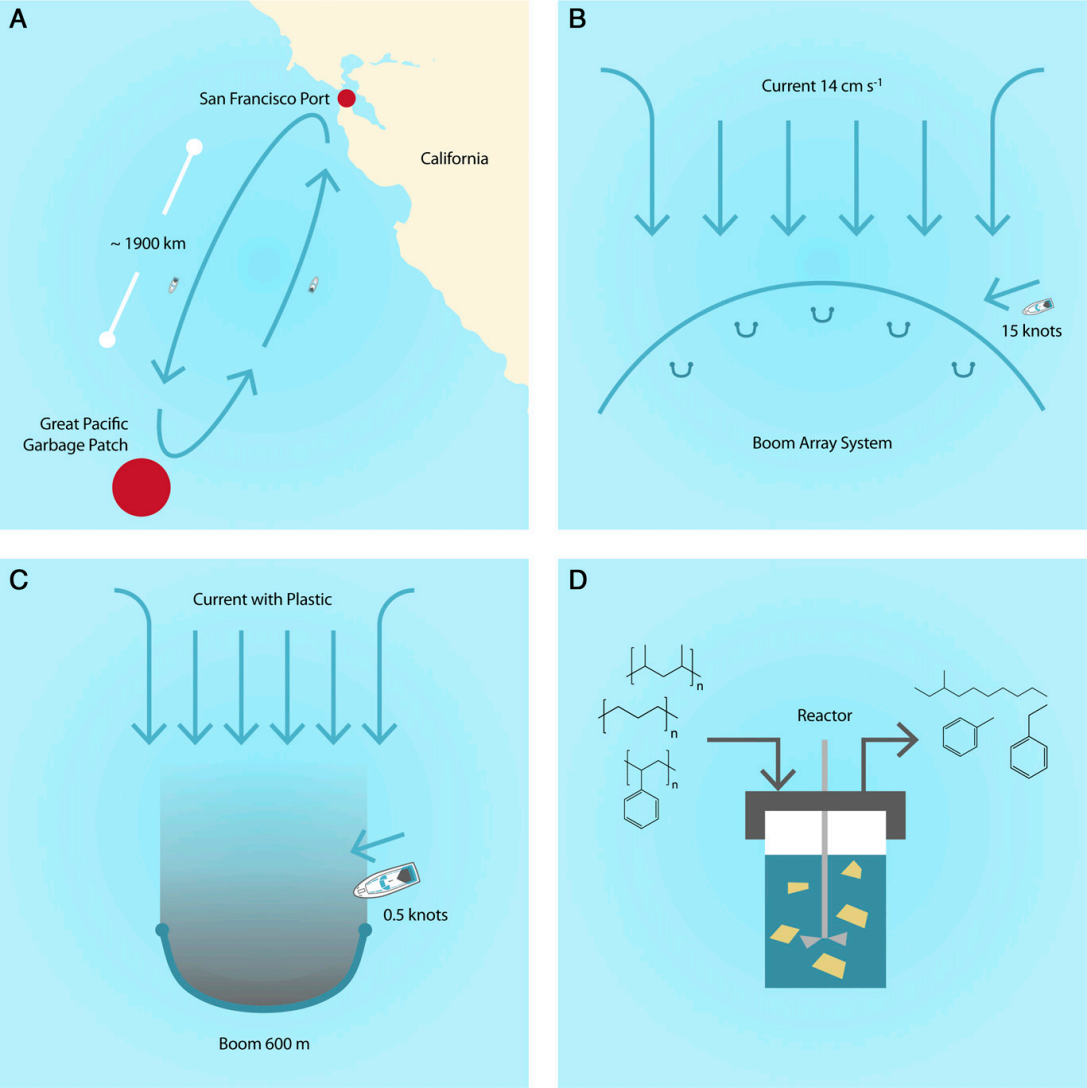
Overview of the process for plastic removal out of the GPGP showing (*A*) the total system overview, (*B*) part of the system of collection booms, (*C*) a single collection boom, and (*D*) the HTL reactor.

Fig. 3*A* locates the port of San Francisco, CA, far from the GPGP. Fig. 3 *B* and *C* shows the deployment of the boom array and the filling of a single boom due to ocean current, respectively, and Fig. 3*D* shows the conversion of plastic into the “blue diesel” replacement fuel. As shown in Fig. 3, the framework requires the following parameters: the size of each boom, the number of booms, the distance between booms, and the distance from the booms to the vessel’s home port.

Existing boom designs (which consist of a series of semicircular floating buoys equipped with a fine mesh extending several feet below the ocean surface) (10) collect plastic at a rate that depends on the loading of incoming plastic, and the speed of the local ocean current, as shown in Fig. 3*C*. Shipboard conversion does not directly change the values of any of these parameters compared with an approach consisting of collecting the plastic, storing it on board, and returning to port to unload. However, shipboard conversion can reduce the frequency of return trips, allowing more booms to be deployed and emptied every year than would be possible with the collect–store–return approach.

Shipboard conversion suggests a second potential advantage over the collection–storage–return approach. While plastic harvesting is typically performed manually to minimize fuel use [with workers manually scooping the plastic out of the booms and into small bags (7)], shipboard conversion makes practical automated plastic collection where the ship can pass through the plastic in the boom and feed it to the conversion process via a conveyor (7). The continuous collection rate is then related to the speed of the ship and the concentration of plastic trapped by the boom. Due to its efficiency, continuous collection was modeled for cases with shipboard conversion. A potential problem with automated scooping is dispersion of the plastic. To minimize wake effects and fuel usage, the ship speed during collection was set to 0.5 knots (kn), and the collection efficiency was set to 70% to account for partial dispersion from the ship’s wake during collection.

The quantity of plastic that could be removed was then calculated for shipboard conversion of plastics to fuel. We assumed the booms are deployed at a distance of 25 km from one another. This distance was selected to minimize boom–boom interactions that would decrease collection efficiency and because 25-km spacing corresponds to the maximum number of booms that can be deployed in the GPGP based on space and be serviced by a single ship in a single year. Details of all of these assumptions and calculations can be found in *SI Appendix*, Table SI.4 and Eqs. SI.1–SI.7.

Using this framework and corresponding assumptions, 2,500 booms could be harvested per year when shipboard conversion is used. A year was assumed to consist of, at most, 240 d, with 16-h days to account for maintenance downtime, weather, sea state, and time off for the crew. Using this value for the number of booms that could be harvested per year and the boom–boom separation distance, the total amount of plastic that could be removed from the GPGP by a single ship yearly at several distinct values of the plastic surface concentration was calculated (1, 2). The results are summarized in Table 2, showing that 1.2 × 10^7^ kg could be removed for the case with the greatest value of plastic surface concentration (2,500 g⋅km^−2^) (1). As plastic surface concentration decreases, the amount of plastic removed decreases, a consequence of the effect of plastic surface concentration on collection in the booms.

**Table 2.** Plastic removed from the GPGP using shipboard HTL for different surface plastic concentrations

Plastic concentration in GPGP* (g⋅km^−2^)	Plastic removed per year^†^ (kg)	Percentage of plastic-derived fuel consumed yearly^‡^ (%)
2,500	1.2 × 10^7^	580
1,000	4.6 × 10^6^	230
500	2.3 × 10^6^	120
200	9.2 × 10^5^	50
50	2.3 × 10^5^	12

* Surface concentration of plastic in the GPGP (1) for a fixed value of 79,000 tons of plastic contained in the GPGP(2).

^†^ Plastic removed from 2,500 booms per year with a 70% collection efficiency.

^‡^ Percentage of total required fuel consumption that can be covered with plastic-derived fuels assuming a fuel density of 0.84 kg/L, a conversion range of 60%, and fuel consumption of 90 GPH.

By eliminating trips to port and by replacing marine diesel with blue diesel, shipboard conversion reduces fuel requirements and, especially, fossil fuel requirements, as shown in Table 2. Specifically, for the highest concentrations, enough plastic can be collected to generate fuel with an excess of 480% that can be stored and used for trips to and from the GPGP, eliminating the need for the use of any fossil fuels. Three of the five concentrations can create an excess of fuel, indicating that areas of low plastic concentration can be supplemented with fuel from higher-concentration areas.

A key assumption in this analysis is that HTL feed rate and conversion rates are equal to the plastic collection rate, so that the ship need not pause periodically to allow time for plastic conversion. This assumption is generally met for the HTL reactor described in the previous section. At the high end of the range of plastic loadings shown in Table 2, the ship will require two to three reactors to match conversion rates with collection rates, which should be easily managed.

Table 2 paints an optimistic picture of GPGP cleanup using shipboard HTL conversion, at least if plastic concentration is present in the higher end of the range of current estimates. On the other hand, if plastic concentrations are on the lower end of the estimated range, cleanup is daunting when considering the current total amount of plastic in the GPGP; the effects of continued accumulation make these estimates even less attractive. Similarly, the size of the ship, or the power rating of the engine, must be carefully selected and its speed controlled, for the process to be thermodynamically favorable. The effect of the composition of the plastic in the GPGP on the thermodynamics of the HTL process adds an extra degree of uncertainty.

## Discussion

Based on the aforementioned considerations, shipboard conversion of oceanic plastic wastes to fuels using HTL is predicted to produce enough exergy to power itself, power the ship, and generate surplus fuel for later use when preconcentrations of plastics via booms are used within a gyre—but not in the open ocean or within a gyre lacking booms. Unfortunately, using the results from Table 2 to calculate cleanup times yields estimates at the edge or beyond what is practical for a single ship to accomplish (7 y to 340 y; see *SI Appendix*, Eqs. SI.1–SI.6 and Table SI.5). Table 2 is based on a 25-km boom–boom separation distance, the distance that corresponds to the maximum number of booms deployed in the GPGP that a single ship can service per year. Accordingly, the framework used to generate estimates in Table 2 was used to evaluate the effects of increasing the number of booms and ships on estimated GPGP cleanup times assuming that no new plastic enters the GPGP during the cleanup operation.

The net effect of decreasing boom–boom separation is to increase the number of booms in the GPGP and hence reduce the cleanup times. The boom–boom separation distance must be sufficient so that upstream booms do not negatively impact the collection efficiency of downstream booms. The minimum distance that avoids the shadow effect is not currently known (24), meaning that boom–boom separation distance can be considered as a parameter in the framework. For a given GPGP area, the boom–boom distance then controls the number of booms deployed and hence the amount of plastic that accumulates—and thereby the estimated cleanup time.

**Fig. 4.**
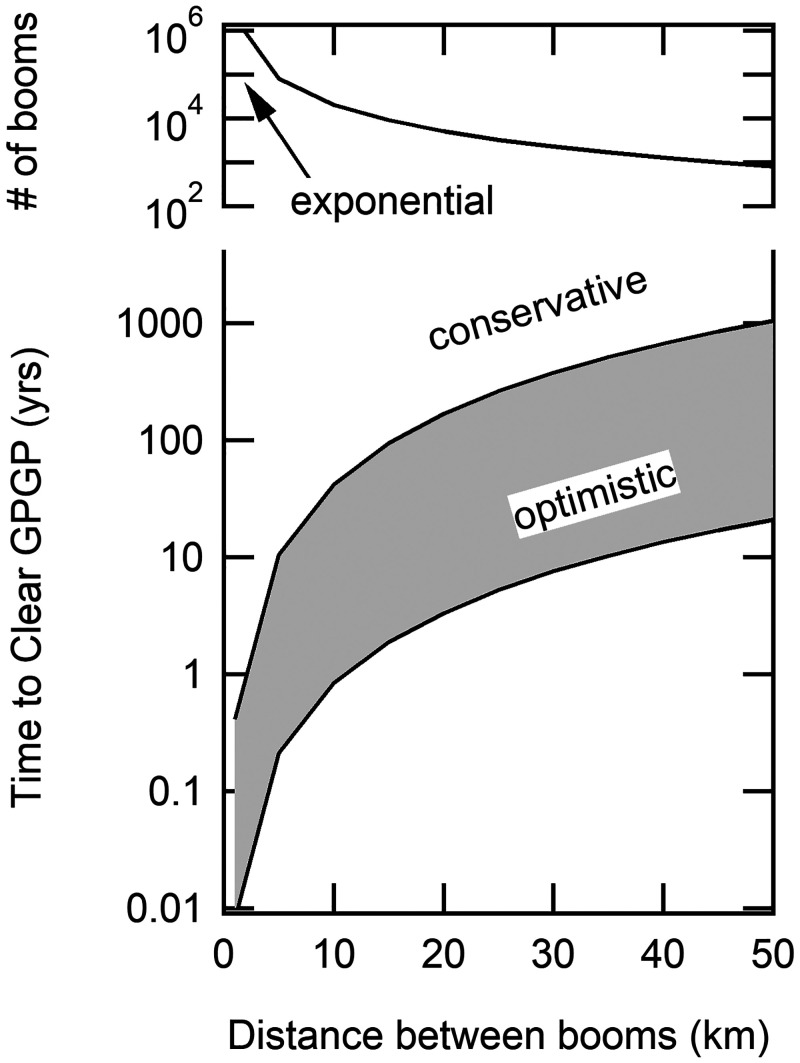
Estimation of the time required to completely clear the GPGP based on high (2,500 g⋅km*^−^*^2^) and low (50 g⋅km*^−^*^2^) concentration estimations (1) for plastic within the GPGP for deployment distance between booms of 1 km to 50 km and the corresponding number of booms deployed.

Fig. 4 shows estimated cleanup times as a function of the distance between booms. Estimates are included for both an optimistic scenario and a conservative one, with the difference being the concentration of plastic currently residing within the GPGP (1). Fig. 4 shows that the time required to clear the GPGP is strongly dependent on the boom–boom distance, with times less than 1 y estimated in the optimistic scenario for distances of ≤10 km. However, Fig. 4 also shows that short cleanup times correspond to huge numbers of deployed booms, a trade-off in cost and practicality that must be considered. Likewise, the boom–boom interactions certainly will impact performance at some separation distance, and this effect should be included in future versions of this framework.

Thermodynamic performance, boom–boom distance, and boom–boom interactions all influence economic and environmental performance. Previous technoeconomic analysis indicates that HTL at a 110 ton/d scale can produce fuels at approximately $4 per gallon of gasoline equivalent (25), a cost that is more than competitive with commercial prices given the fuel savings predicted for self-powered cleanup. More appropriately for the shipboard application, a rigorous, stochastic economic performance assessment, based on published analysis of a similar system and outlined in *SI Appendix*, indicates that the capital cost of the modular conversion system of the appropriate scale (10 ton/d) would be $2.85 million to $3.52 million with operating costs of $890,000 to $990,000. These estimates do not include the savings associated with reduced fuel purchases, meaning that the marginal cost of the conversion system is therefore much less than the estimated cost of the boom system and ship itself (8).

The cleanup times estimated here, which are constrained by the number of booms that can be deployed in the GPGP, either are similar in magnitude to environmental degradation timescales (6) or require thousands of deployed booms. Given the cleanup timescales and costs, efforts might be better placed on interception at locations of high plastic flux to prevent plastic reaching the patches in the first place (9). River mouths are one potential location where a boom and collection system could be more strategically deployed than at the oceanic gyres (9). Similarly, boom systems might be placed to protect especially fragile or valuable ecosystems, such as breeding grounds. Alternative approaches involving mobile collection technologies are possible, but these options do not appear to reduce collection times sufficiently to justify the greater technological complexity. More details are provided in *SI Appendix*.

The current analysis shows that collection of plastics that have already entered the environment is, at most, part of a multilayered approach to mitigate environmental damage from plastics. Other key elements include reducing plastic use (26), improving the recyclability of plastics, or increasing the biodegradability of plastics (27). Finally, it should be pointed out that implementing a plastic waste harvesting technology, whether it is based on HTL, pyrolysis, or some other technology option, would require further study and enhanced understanding of attendant issues, including unavoidable spills (28) and engine performance and emissions characteristics (29), as well as fuel delivery system integrity (30) when running on the plastic-derived fuel.

This analysis has focused on HTL as a technologically deployable method suitable for plastic conversion. However, the results presented here can reasonably be extended to other conversion methods. Enzymatic conversion (31, 32), which has been the subject of several recent studies, could be especially attractive for conversion at low temperatures, thereby improving thermodynamic efficiency and reducing hazards to the crew. Since HTL already produces sufficient energy to power the process and the ship, the thermodynamic benefit of enzymatic conversion will be incremental rather than transformative. Similarly, enzymatic conversion rates are slower than HTL rates, meaning that larger reactor vessels are required for enzyme-based conversion reactions than required for HTL (31). Accordingly, the transformative impact of the enzymatic conversion would be for degradation of the plastics into harmless products in the ocean without harvesting them. The current analysis indicates that research of in situ enzymatic decomposition of plastics to harmless products is warranted.

Converting marine plastics into fuel will ultimately release the carbon they contain as greenhouse gas emissions. That stated, the quantities of released CO_2_ are a small percentage of the global emissions budget, currently ∼485 PgC (33). If the system ran continuously for 10 y, the total percentage would be less than 0.02% of the global carbon budget. On the other hand, converting the plastic into fuel eliminates new fossil emissions while simultaneously cleaning the oceans and reducing the amount of plastic being recycled in land-based operations. Converting the plastic to fuel also eliminates unnecessary congestion at ports and reduces the chance of a nearshore oil spill. Unlike petroleum fuels, plastic-derived fuels have low sulfur content, meaning that their combustion will not release sulfur oxides (34, 35), which is a desirable outcome given the importance of SO_2_ in the formation of pollutants (36) and new regulations limiting sulfur content in fuels (37).

The results of this work provide a strong argument for continued study to advance current understanding of the factors that affect marine plastic depolymerization in real systems. Advances in the scientific knowledge of depolymerization thermodynamics, rates, and product distributions of ocean-borne plastics are required to reduce uncertainty around the HTL approach. Similarly, the cleanup framework can be improved by filling gaps in current estimates of marine plastic concentrations, quantities, and fluxes to reduce uncertainties in deployment outcomes. Finally, the results of this work show the immense challenge facing the prospect of cleaning up the ocean and argue for the need to change current plastic use and recycling strategies. Accordingly, the sound probability analysis and framework presented here can be incorporated with risk analysis to insightfully and reliably inform future decision-making and policy responses in this important area.

In summary, accumulation of waste plastics in the world’s oceans is a pressing problem that demands commensurate attention. Cleanup that relies on returning the plastics to port will be fuel intensive. The plastic itself contains chemical energy that can offset petroleum consumption and potentially reduce cleanup times by reducing the number of times the cleanup vessel must return to port to unload and refuel. This work shows that HTL should be able to provide the exergy required for self-powered cleanup, provided that surface plastic concentrations of >12 vol% are available. Estimated cleanup times then depend on the rate at which plastic accumulates in collection booms, with cleanup times decreasing with the number of booms deployed. Economically, the HTL system is a modest additional cost relative to the cleanup vessel and boom system. Based on this promising analysis, future work can evaluate effects of additives, contaminants, and aging on HTL oil yields and the suitability of plastic-derived fuels for existing fuel delivery systems and engines.

## Methods

### @Risk Model.

All the uncertain model input variables were modeled using appropriately selected uniform distributions in the absence of any accumulated operating experience and pertinent historical data. MC simulation runs were conducted using the @Risk software (38) with 10,000 iterations for each volume percent of plastic in the inlet stream.

### Process Details.

*SI Appendix*, Table SI.1 includes a list of the equipment and their energy requirements. Salt is removed from the feed to protect the reactor and other materials from corrosion. Residual salt is removed using a reverse osmosis system (operating at 99% efficiency) to reduce the salt concentration in the system to levels that are compatible with high-grade stainless steel (<1 wt %) (39). To increase the effectiveness of depolymerization and permit feeding to the reactor, the plastics must be shredded at the process intake (40, 41). *SI Appendix*, Table SI.2 shows reaction conditions met by the equipment for the plastics studied. The shredded plastic is then combined with desalinated water to 10 wt % to 30 wt % solids, preheated in the heat exchanger, pumped to pressure, and heated to reaction temperature. The stream exiting the reactor is cooled to form organic and water-rich products (11–13) which are separated in a gravity separator. Residual organics in the aqueous phase are removed in a hydrogen peroxide/ultraviolet (UV) oxidation system prior to recycling. The HTL product oil (composition shown in *SI Appendix*, Table SI.3) is fed to an engine, to generate power, operating at an efficiency of 35 to 40%.

### Exergy Calculations.

The exergy of each subprocess was individually calculated and then summed and normalized based on the mass of plastic in the inlet.

### Chemical Exergies and Combustion.

The chemical exergies and exergy of combustion of the stream were calculated as the weighted average of the Gibbs free energy of each product including water, at standard temperature and pressure and steady state and the weighted average based on the enthalpy of combustion and their published yields, respectively. Thermodynamic data for pure plastics and products were taken from the literature (42–45) and National Institute of Standards and Technology (46), and yields were found in the pertinent literature (11–13). When the thermodynamic values (enthalpy and entropy) of the products could not be found, correlations based on carbon number were used to estimate them (see *SI Appendix*, Eqs. **SI.8**–**SI.13**). It was assumed that 100% of the oil formed from HTL could be directly combusted without upgrading.

### UV/Hydrogen Peroxide System.

A UV subprocess was modeled for organic removal using a 300-nm lamp. The solubility values of the products of polystyrene in water were found in the pertinent literature (47–50). Further details of this calculation can be found in *SI Appendix*, section SI.4.

## Data Availability

All study data are included in either the *SI Appendix* or online at Digital WPI (https://digital.wpi.edu/show/8c97kt62t).
